# Adverse events associated with 1540-nm nonablative fractional resurfacing in darker skin: A retrospective study

**DOI:** 10.1016/j.jdin.2023.10.010

**Published:** 2023-11-07

**Authors:** Henriette De La Garza, Michelle Lazar, Poom Visutjindaporn, Neelam A. Vashi

**Affiliations:** a Department of Dermatology, Boston University School of Medicine, Boston, Massachusetts

**Keywords:** adverse events, ethnic skin, hyperpigmentation, laser, scarring, skin of color

*To the Editor:* Laser resurfacing has been difficult historically in people with darker skin tones given the increased risks of adverse events (AEs). Available treatments may incite further pigment alteration and scarring. Nonablative fractionated resurfacing, such as 1540/1550-nm technology, is used for a myriad of treatments, including rejuvenation, dyschromia, scar reduction, and blemish removal.^1^ Early findings show favorable outcomes in people with Fitzpatrick skin types (FSTs) IV to VI.^2^^,^^3^ The purpose of this study was to further evaluate and characterize the efficacy and safety profile of nonablative fractional 1540-nm laser treatment in patients with darker skin tones.

A 10-year, single-center, retrospective analysis of adults who received at least 1 treatment with the nonablative fractional 1540-nm laser was conducted to determine the frequency of AEs in patients with FSTs I to VI. AEs are reported standardly in chart notes at this institution. All treatments were performed by board-certified dermatologists with extensive laser training and experience. A total of 1160 patients were treated with the 1540-nm modality during the study period. The average treatment had 277 pulses, had a pulse duration of 14.88 seconds, and required 2.9 passes to complete. Of the 1160 treatments included in the analysis, 140 (12.07%) were associated with an AE (Table I). The most frequently reported AEs were extended redness (91, 7.84%), swelling (28, 2.41%), and crusting (17, 1.47%), with the majority occurring on the face (130, 11.21%) (Table II). The most common indications for all treatments were scar (81.56%), dyschromia (8.10%), and striae (5.86%). Of these treatments, scar treatment was associated with the highest rate of total AEs (13.31%). Patients aged from 35 to 44 years (19.00%) and 45 to 54 years (16.96%) had the highest percentage of AEs. Furthermore, there was a statistically significant difference in the mean pulse count between those who experienced AEs (mean pulse count, 240.86) and those who did not (mean pulse count, 182.16; *P* = .0010). The majority of patients (69.13%) considered their treatment helpful and reported an improvement in their condition.

**Table I:** Demographic information

Population characteristics	Total cohort (%), *n* *=* 1160	No adverse events (%), *n* *=* 1015	Adverse events (%), *n* *=* 140	Not reported		
Race						
American Indian	41 (3.53)	34 (82.93)	7 (17.07)	0		
Asian	189 (16.29)	176 (93.12)	13 (6.88)	0	Race	*P* value
Black/African American	117 (10.09)	111 (94.87)	4 (3.42)	2	White vs Black	.0024†
Hispanic or Latino	103 (8.8)	77 (74.76)	26 (25.24)	0	White vs Asian	.0276∗
Middle Eastern	29 (2.50)	29 (100.00)	0 (0.00)	0	White vs Hispanic	.0036†
Other, Pacific Islander	4 (0.34)	4 (100.00)	0 (0.00)	0	Black vs Asian	.3041
Other	20 (1.72)	18 (90.00)	2 (10.00)	0	Black vs Hispanic	<.0001‡
White	454 (39.14)	393 (86.56)	59 (13.00)	2	Asian vs Hispanic	<.0001‡
Declined	203 (17.50)	173 (85.22)	29 (14.29)	1		
Gender						
Male	406 (35.00)	376 (92.61)	30 (7.39)	0		
Female	754 (65.00)	639 (84.75)	110 (14.59)	5		
FST						
I	5 (0.43)	2 (40.00)	3 (60.00)	0		
II	303 (26.12)	264 (87.13)	37 (12.21)	2		
III	203 (17.50)	185 (91.13)	18 (8.87)	0		
IV	174 (15.00)	146 (83.91)	28 (16.09)	0		
V	97 (8.36)	87 (89.69)	9 (9.28)	1		
VI	28 (2.41)	26 (92.86)	2 (7.14)	0		
Not applicable	350 (30.17)	305 (87.14)	43 (12.29)	2		

All analyses were conducted using Prism 9 GraphPad Software via Fischer’s exact test.

∗ *P* < .05

† *P* < .01

‡ *P* < .001.

**Table II:** Adverse event characteristics

Location of AEs	AE present	AE absent	Unknown status AE	% of total reported AEs				
Face	130	816	5	13.67%				
Trunk	2	68	0	2.94%				
Extremities	7	81	0	8.64%				
Most common AEs	Total count	FSTs I and II	FSTs III and IV	FSTs V and VI	Unknown FST	AE absent	Unknown	% of specific indication
Redness	91	33	24	8	26	73	0	5.19%
Swelling	28	9	15	0	4	801	5	13.52%
Crusting	17	5	9	1	2	61	0	8.96%
Hyperpigmentation	13	-	-	-	-			
Acne	13	-	-	-	-			
Scabbing	8	-	-	-	-			
Indications for treatment	AE present	Laser settings	Mean pulse count	Mean pulse duration				
Dyschromia	4	AE present	240.86	15.00				
Scar	126	AE absent	182.16	14.87				
Striae	6							

Black (3.42% Black vs 13.00% White; *P* = .0024), Asian (6.88% Asian vs 13.00% White; *P* = .0276), and Hispanic (25.24% Hispanic vs 13.00% White; *P* = .0036) patients had statistically significantly different rates of AEs compared with White patients based on the assessment by Fisher’s exact test. Differences in the rates of AEs may be related to higher energy used in patients with FSTs I to III, provider-dependent differences, prior retinol use, and/or use of overall more conservative parameters (ie, lower pulse count and less overlap) in some groups.

The strength of this study is the diversity of patients, both in terms of FST and race/ethnicity. The limitation lies in the single-center design, which may limit generalizability. We hope to add to the body of literature that can provide evidence-based support for protocol determination, patient education, and continued training and research on cosmetic treatments in patients with darker skin tones. In dermatologists with trained expertise, the 1540-nm nonablative fractional laser treatment is an effective and safe method for improving scar, dyschromia, and striae in all skin types.

## Conflicts of interest

None disclosed.
